# Clinical manifestations and outcomes of otitis media with effusion in adult patients following Omicron infection in China

**DOI:** 10.17305/bb.2024.10239

**Published:** 2024-08-01

**Authors:** Xiangming Meng, Ying Wang, Chengzhou Han, Xiaobo Gu, Chao Hang, Jianxun Guo, Yuting Jiang

**Affiliations:** 1 Department of Otolaryngology, Affiliated Huishan Hospital of Xinglin College, Nantong University, Wuxi Huishan District People’s Hospital, Wuxi, China; 2 Department of Otolaryngology, Wuxi Huishan District Qianqiao Street Community Health Service Center, Wuxi, China

**Keywords:** Coronavirus disease 2019 (COVID-19), otitis media with effusion (OME), severe acute respiratory syndrome coronavirus 2 (SARS-CoV-2), Omicron, zero-COVID-19 policy.

## Abstract

Within the constantly changing landscape of the coronavirus disease 2019 (COVID-19) pandemic, the emergence of new variants introduces novel clinical challenges, necessitating the acquisition of updated insights into their impacts on various health conditions. This study investigates the clinical features and therapeutic outcomes of otitis media with effusion (OME) in adults following infection with the Omicron variant of COVID-19, in the context of China ending its “Zero-COVID-19” policy. Conducted as a multicenter, retrospective analysis at two medical institutions in Eastern China from December 2022 to February 2023, the study included patients with confirmed Omicron infection who were diagnosed with OME within two months, adhering to guidelines from the American Academy of Otolaryngology-Head and Neck Surgery Foundation (AAO-HNSF). Data on demographics, time from infection to OME manifestation, associated symptoms, and treatment outcomes were collected. Among 68 patients (73 affected ears) with OME post-Omicron infection, common symptoms included cough and nasal obstruction (69.1%). All reported ear fullness, with 86.8% experiencing hearing loss. Tympanic bullae were observed in 72.6% during otoscopy, and most tympanometry results showed a B-type tympanogram (80.0%). An integrated treatment strategy led to an 83.6% cure rate, although 8.2% experienced relapse within 2–3 months. Our findings highlight OME as a prevalent ear complication associated with COVID-19 during the Omicron pandemic, underscoring the necessity for further investigation into its complexities. While the integrated treatment approach proved effective, the 8.2% post-treatment recurrence rate underscores the importance of ongoing monitoring and signals an urgent need for more comprehensive research.

## Introduction

The coronavirus disease 2019 (COVID-19) is caused by severe acute respiratory syndrome coronavirus 2 (SARS-CoV-2), a highly transmissible and pathogenic human coronavirus [1]. The COVID-19 pandemic has been ongoing globally for over three years, resulting in many infections and a profound impact on public health. Initially, respiratory symptoms such as cough, fever, and shortness of breath were considered characteristic manifestations of COVID-19 during the early phase of the outbreak [2]. However, as the COVID-19 pandemic continues to evolve, increasing evidence suggests that SARS-CoV-2 infection may be associated with otolaryngological conditions, including anosmia, sudden sensorineural hearing loss, and acute epiglottitis [3–6]. Furthermore, maternal COVID-19 infection may have implications for the hearing of newborns [7].

Otitis media with effusion (OME) is characterized by fluid in the middle ear (ME) without the signs and symptoms of acute otitis media [8]. OME is highly prevalent among preschool-aged children, affecting at least 90% of patients and ranking as one of the primary causes of hearing loss [9]. The pathogenesis of OME in adults is potentially linked to various factors, including eustachian tube dysfunction, gastroesophageal reflux disease, laryngopharyngeal reflux, nasal polyposis, the levels of vascular endothelial growth factor, nasopharyngeal carcinoma, and smoking [10–15].

Upper respiratory tract infections are widely acknowledged as significant precipitating factors in the development of OME [8, 9]. Throughout the COVID-19 pandemic, preventive and control strategies significantly impacted the incidence of OME [16, 17]. The development of OME may be associated with inflammatory stimuli, including bacteria, viruses, and allergies [18]. A recent investigation examining ME secretion samples from OME patients revealed the presence of various respiratory viruses, such as adenovirus, influenza B, rhinovirus, bocavirus, parainfluenza 4, coronavirus OC43, and respiratory syncytial virus A/B [19]. These respiratory viruses may contribute to the pathogenesis of OME.

Recent studies have confirmed the presence of SARS-CoV-2 in the ME effusion (MEE) of patients with OME post-COVID-19 infection, highlighting the virus’s critical role in the pathogenesis of OME [20, 21]. Additionally, SARS-CoV-2 is known to exhibit a high viral load in the nasopharynx [22]. The ACE2 receptor, which acts as the cellular entry point for SARS-CoV-2, shows high expression in the ciliated cells of the eustachian tube, potentially facilitating the virus’s spread to the ME [23].

China has consistently adhered to the “Zero-COVID-19 policy,” effectively preventing the infection of many individuals. However, since December 7, 2022, when China discontinued this policy, there has been a sharp increase in SARS-CoV-2 infections [24, 25]. During this wave of the pandemic in China, the prevailing Omicron variants were BA.5.2 and BF.7 [25]. There was a significant increase in outpatient OME cases following SARS-CoV-2 infection [20]. However, our knowledge regarding OME following SARS-CoV-2 infection is limited.

Previous research showed age influences the duration of viral RNA shedding in COVID-19, with older patients experiencing prolonged shedding [26]. Therefore, understanding the pathophysiology, clinical presentation, and management of OME in adult patients post-Omicron infection is crucial. This study aims to examine the clinical characteristics and treatment outcomes of OME in adult patients who followed the Omicron infection in light of China’s decision to end its “Zero-COVID-19 policy”.

## Materials and methods

### Patients

This multicenter retrospective study was conducted from December 2022 to February 2023 in the otolaryngology departments of two medical institutions in Eastern China. One of the institutions involved in the study is Wuxi Huishan District People’s Hospital (WHDPH), a tertiary hospital, while the other is Qianqiao Street Community Health Center (QSCHC), a primary healthcare institution. These medical institutions are part of the same medical consortium [27].

This study performed a retrospective analysis by examining electronic medical records of patients diagnosed with OME following infection with the Omicron variant of SARS-CoV-2. The identification of cases was facilitated using the ICD-10 code H65.9. The review process, spanning June and July 2023, involved methodical extraction and organization of data into a predefined Microsoft Excel spreadsheet. Patients were enrolled in the study if they met the following inclusion criteria: 1) a confirmed diagnosis of SARS-CoV-2 infection, verified by a positive COVID-19 antigen test result following the 10^th^ edition of the Diagnostic and Treatment Guidelines issued by the National Health Commission of China [28], 2) a diagnosis of OME within two months following infection, determined based on the Clinical Practice Guidelines by the American Academy of Otolaryngology—Head and Neck Surgery Foundation (AAO-HNSF) [8], 3) having received treatment at one of the specified medical institutions, and 4) possession of complete medical records. The exclusion criteria were a history of chronic otitis media, autoimmune diseases, previous ear surgery, or loss of follow-up.

Hearing thresholds of patients were evaluated through audiometric testing, employing an audiometer (Model 1066, Otometrics A/S, Denmark). The evaluation occurred in a standard soundproof chamber where air and bone conduction pure-tone thresholds were determined using an ascending–descending method. Testing commenced with the better ear, followed by the affected ear, prioritizing air conduction over bone conduction across frequencies from 0.25 to 8 kHz. Retest frequencies were set at 1 kHz, with subsequent tests at 0.5 and 0.25 kHz. Bone conduction testing was confined to octave frequencies ranging from 0.25 to 4 kHz. Additionally, an impedance audiometer (Model 901, Otometrics A/S, Denmark) was utilized to conduct tympanometry, assessing the patients’ acoustic impedance.

We gathered various types of data for this study. These included demographic characteristics such as age and gender, as well as the side of the ear affected. We also considered the time interval between the onset of infection with the Omicron variant and the manifestation of OME. Additionally, we recorded symptoms associated with the Omicron infection, encompassing cough, fever, sore throat, nasal obstruction, and runny nose. Clinical features were noted, including the duration of symptoms and specific auditory complaints like hearing loss, ear fullness, tinnitus, and ear pain. Treatment approaches were documented, including medication, tympanocentesis, and auditory massage. Finally, we conducted audiological examinations and tracked treatment outcomes, categorizing them as “recovered,” “improved,” or “untreated,” followed by a follow-up period.

### Ethical statement

This study was approved by the Ethics Committee of Wuxi Huishan District People’s Hospital (Grant number: HYLL20230601001). It was conducted strictly with the ethical principles outlined in the Helsinki Declaration and any subsequent amendments and other relevant ethical standards. All patients in this study provided informed consent to publish their clinical data and images in the manuscript.

### Statistical analysis

The statistical analysis was performed using R software (version 4.2.2). Descriptive statistics were utilized to summarize the findings. Categorical variables were presented as frequency counts (*n*) and corresponding percentages (%). The normality of continuous variables was assessed through histograms and QQ plots. Variables conforming to a normal distribution were described using mean and standard deviation (SD) to depict their central tendency and variability precisely. In contrast, medians and interquartile ranges (IQR) were utilized for those variables diverging from normal distribution, as these measures more aptly characterize the central tendency and dispersion of non-normally distributed data.

## Results

A total of 68 patients diagnosed with OME following Omicron infection were included in the study. The cohort comprised 31 males (45.6%) and 37 females (54.4%). Among them, 34 (50.0%) experienced left-sided onset, 29 (42.6%) had right-sided onset, and 5 (7.4%) showed bilateral onset. The mean age of these patients was 50.9 years, with an SD of 16.4 years. The mean age was 50.9 years, with an SD of 16.4 years. The median interval from Omicron infection to OME diagnosis was 8.5 days, with an IQR of 5.0–14.3 days. The median duration of OME symptoms was 7.0 days (IQR 6.0–13.3 days). Detailed patient characteristics are presented in Table 1.

**Table 1:** Patient demographics and baseline characteristics

**Characteristics**	
Gender	*n* (%)
Male	31 (45.6)
Female	37 (54.4)
Age (years)	Mean ± SD
	50.9 ± 16.4
Sides	*n* (%)
Left	34 (50.0)
Right	29 (42.6)
Bilateral	5 (7.4)
Interval between Omicron infection and OME diagnosis (days)	Median (IQR)
	8.5 (5.0, 14.3)
Duration of OME symptoms (days)	Median (IQR)
	7.0 (6.0, 13.3)
Pure tone audiometry (*n* ═ 40)*	Mean ± SD
	33.4 ± 15.7 dB nHL
Air-bone gap (*n* ═ 40)*	Mean ± SD
	10.5 ± 8.5 dB nHL
Tympanometry (*n* ═ 40)*	*n* (%)
B-type	60 (82.2)
C-type	9 (12.3)
A-type	4 (5.5)

*38 patients with 40 affected ears. SD: Standard deviation; OME: Middle ear effusion; IQR: Interquartile range.

**Figure 1.**
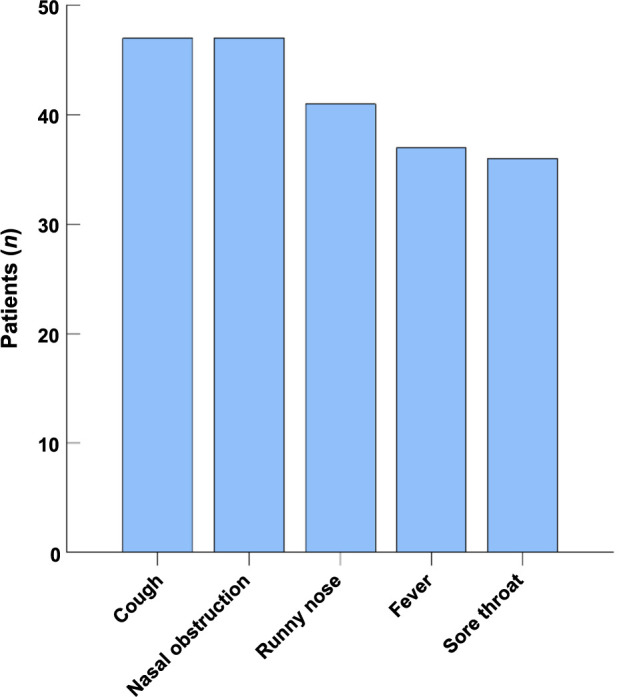
**The distribution of symptoms presented by patients following Omicron infection**.

**Figure 2.**
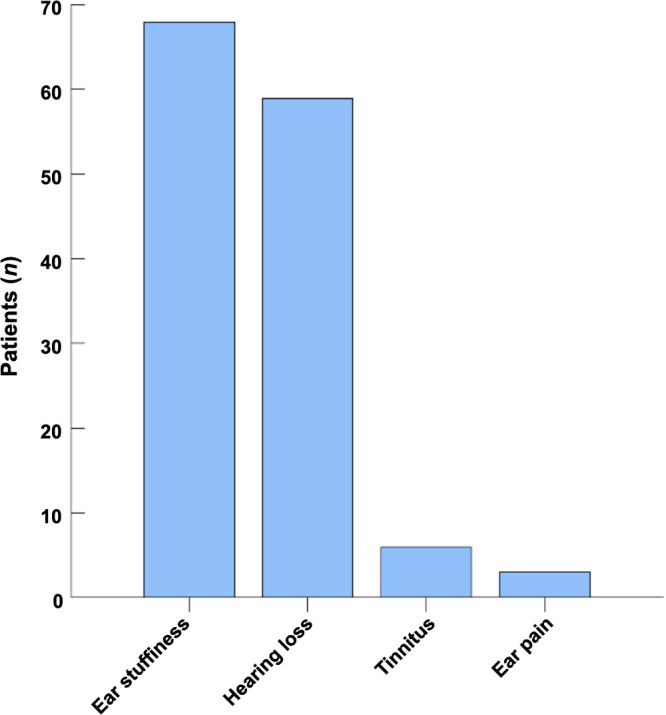
**The distribution of symptoms exhibited by patients with otitis media with effusion following Omicron infection**. OME: Otitis media with effusion.

The most frequently observed symptoms among patients infected with the Omicron variant were cough and nasal obstruction, affecting approximately 47 (69.1%) patients. Other common symptoms included a runny nose, fever, and sore throat, observed in 41 (60.3%), 37 (54.4%), and 36 (52.9%) patients, respectively. Figure 1 illustrates the distribution of these symptoms. Ear stuffiness was the predominant symptom in all 68 (100%) patients diagnosed with OME. Hearing loss was also a common complaint, reported by 59 (86.8%) patients. Tinnitus and ear pain were reported less frequently in six (8.8%) and three (4.4%) cases, respectively. Figure 2 provides an overview of symptom distribution within the OME patient cohort.

**Figure 3.**
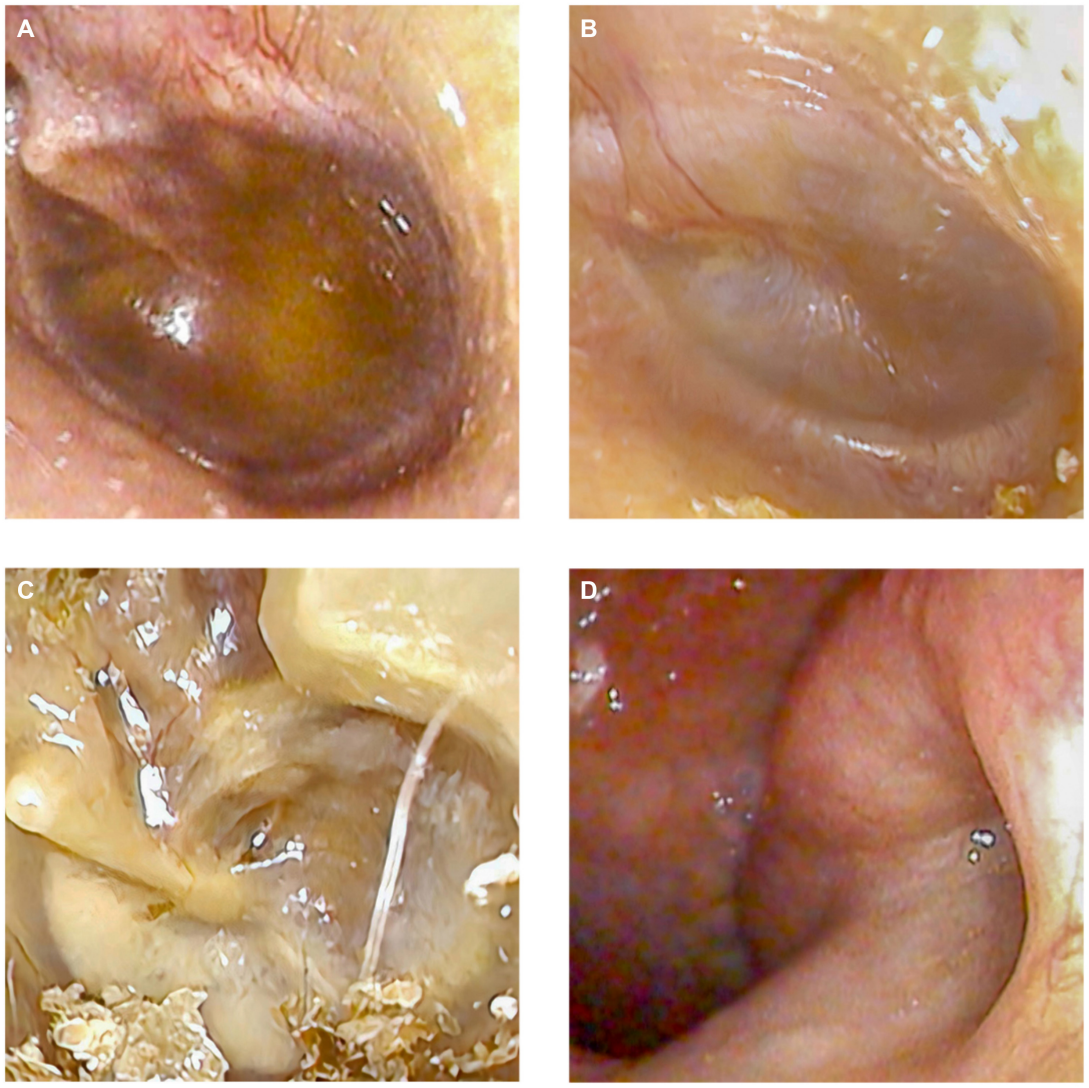
**The tympanic membrane and the pharyngeal ostium of the eustachian tube in patients diagnosed with otitis media with effusion following Omicron infection.** (A) Yellowish fluid accumulation in the left middle ear; (B) Congestion of the tympanic membrane in the left middle ear; (C) Invagination of the tympanic membrane in the left middle ear; (D) An obstructed pharyngeal ostium of the left eustachian tube, accompanied by surrounding mucosal edema.

All patients diagnosed with OME underwent otoscopy, bulging tympanic membranes, and yellowish fluid in the ME were observed in 63 ears (86.3%), as depicted in Figure 3A. Additional otoscopic observations included tympanic membrane congestion in six (8.2%) ears and invagination in four (5.5%) ears, respectively, illustrated in Figure 3B and 3C. Furthermore, high-resolution electronic nasopharyngoscopy performed on 14 patients (20.6%) revealed obstructed pharyngeal ostia of the Eustachian tubes and surrounding mucosal edema, as shown in Figure 3D.

**Figure 4.**
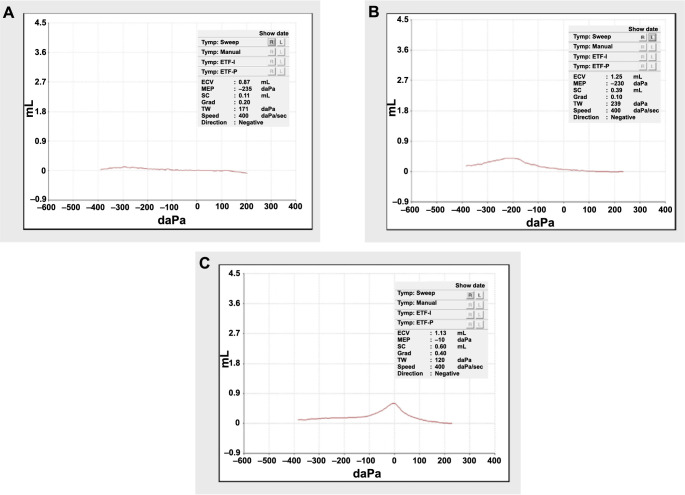
**The various types of tympanometry results in patients with otitis media with effusion following Omicron infection.** (A) B-type tympanogram; (B) C-type tympanogram; (C) A-type tympanogram.

Among the patients, 38 (covering 40 ears) received comprehensive audiological evaluations, including tympanometry and pure-tone audiometry (PTA). Tympanometry results indicated negative ME pressure and reduced compliance. Among the 40 ears analyzed, 32 (80.0%) exhibited a B-type tympanogram (Figure 4A), 6 (15.0%) a C-type (Figure 4B), and 2 (5.0%) an A-type tympanogram (Figure 4C). PTA results showed an average threshold of 33.4 ± 15.7 dB nHL and an average air-bone gap of 10.5 ± 8.5 dB nHL.

Every patient was treated with oral antibiotics, mucolytics, nasal decongestants, and nasal corticosteroids, supplemented by daily auditory massages. In our study, tympanocentesis was administered to 18 ears (24.7%). Following the procedure, significant hearing improvements were noted in all these cases, absent any complications.

After receiving therapeutic interventions, comprising medication and tympanocentesis, most OME patients experienced symptom relief. Specifically, out of the total ears examined, 61 (83.6%) were deemed cured, 10 (13.7%) improved, and 2 (2.7%) remained uncured. A follow-up, spanning 2–3 months, was conducted for all these patients. It was observed that six ears (8.2%) encountered symptom recurrence roughly two months after the intervention.

## Discussion

This study assessed the clinical features and treatment outcomes of adult Chinese patients diagnosed with OME after infection by the Omicron variant. Our results indicated that post-Omicron infection, cough, and nasal obstruction were predominant symptoms reported by 69.1% of the study participants. The median duration from the onset of Omicron infection to OME diagnosis was 8.5 days (IQR 5.0–14.3). Notably, all patients reported experiencing ear stuffiness as a symptom of OME, while 86.8% of the cohort reported hearing loss.

Our findings indicate that following comprehensive treatment, 83.6% of patients achieved complete remission. Conversely, Fan et al. [29] observed in their cohort study that only 40.7% achieved complete remission, while 51.9% showed improvement, and 7.4% experienced no change. This variation could be attributed to differing population characteristics. The research by Zhong et al. [30] demonstrated that intranasal steroids are effective in treating adult patients with OME. Liang et al. [23] proposed that administering steroids via intratympanic injection is an optimal approach for minimizing systemic adverse effects. Zhang et al. [31] reported that tympanocentesis has a beneficial therapeutic effect for these patients, as it aids in the clearance of SARS-CoV-2 in the MEE. It is essential for the medical process to consider the treatment effectiveness and its environmental impact, particularly in the context of the COVID-19 pandemic [32].

The following factors may contribute to SARS-CoV-2 infection-related OME. First, upper respiratory tract viral infections trigger an inflammatory reaction in the nasopharynx, which might ultimately result in eustachian tube blockage. Zhang et al. [31] observed that all patients with OME caused by COVID-19 exhibited Eustachian tube dysfunction and considered it a fundamental basis and pathway for OME development. Second, the Omicron variation of SARS-CoV-2 generates a higher and more sustained viral load in the nasopharynx than the previously predominant Delta version [33]. Third, SARS-CoV-2 might invade the ME mucosae and the mastoids [21]. Fourth, the patient experienced nasal congestion and a runny nose during the SARS-CoV-2 infection. In this wave of Omicron variant infections, patients had more severe nasal symptoms, referred to as “cement nose” in China. Inappropriate nose-blowing techniques among a minority of patients may have contributed to the emergence of OME.

In the recent Omicron infection wave, predominant symptoms included upper respiratory manifestations, such as coughing, nasal obstruction, and rhinorrhea. Consequently, our therapeutic strategy was adapted to offer symptomatic relief, integrating nasal decongestants and corticosteroids to target nasal symptoms specifically. Considering the potential inflammatory response in the nasopharynx induced by the Omicron variant, patients were also administered oral antibiotics. These interventions proved highly effective for OME treatment, with only 18 ears (24.7%) necessitating tympanocentesis. However, the recurrence of OME in six ears two months after clinical resolution was a notable observation. This recurrence might be linked to the extended presence of SARS-CoV-2 in the MEE [20]. Recent studies have shown that SARS-CoV-2 remains in the MEE longer than in the nasopharynx and oropharynx [23]. Specifically, Fan et al. [29] documented that SARS-CoV-2 can persist in the MEE for up to 45 days after a patient receives a negative nasopharyngeal swab result. It indicates that a negative nasopharyngeal or oropharyngeal swab test does not conclusively confirm the absence of SARS-CoV-2 in the body, underscoring the virus’s ongoing infectious risk.

Our study acknowledges several limitations that are important to consider when interpreting its results. First, its retrospective design and small sample size may impact the generalizability of our findings. Many participants’ reliance on self-administered SARS-CoV-2 antigen tests introduces a potential for interpretation errors, which could affect the accuracy of COVID-19 diagnoses. Additionally, the absence of specific subtyping for the Omicron variant limits our ability to compare outcomes across different Omicron subtypes, potentially affecting the study’s applicability to broader populations. A significant limitation was the unavailability of audiograms for nearly half of the patients with OME, primarily due to widespread infections among healthcare workers during the Omicron outbreak. This shortfall could limit the depth of our audiological insights. Furthermore, the MEE in patients with OME was not tested for SARS-CoV-2, rendering the virus’s presence in the ME uncertain. Lastly, the prevalence rate of OME diagnoses following Omicron infection remains ambiguous, underscoring the need for further research. A comprehensive survey involving a substantial cohort of Omicron-infected individuals would be instrumental in shedding light on these aspects. In future research, we aim to conduct SARS-CoV-2 testing on the MEE in patients with OME. These limitations collectively highlight areas for future study and consideration in interpreting our current findings.

## Conclusion

Our study indicates that OME was among the most common ear complications associated with COVID-19 and ME viral infections during the Omicron pandemic. Although an integrated treatment strategy proved effective, the 8.2% rate of recurrence post-treatment emphasizes the need for continuous monitoring. However, the retrospective design and small sample size of our study constrain our capacity to ascertain causality definitively. Therefore, future investigations, encompassing rigorously controlled trials and longitudinal studies on the viral presence in the MEE, are imperative to elucidate the relationship between COVID-19 and OME definitively and to enhance clinical management practices. Such research will deepen our comprehension of the pathogenesis of OME in the context of COVID-19 and guide the development of screening and monitoring protocols to address the long-term auditory consequences.
